# Defect localization by an extended laser source on a hemisphere

**DOI:** 10.1038/s41598-021-94084-w

**Published:** 2021-07-26

**Authors:** Daniel Veira Canle, Joni Mäkinen, Richard Blomqvist, Maria Gritsevich, Ari Salmi, Edward Hæggström

**Affiliations:** 1 Department of Physics, Division of Material Physics, Faculty of Science, University of Helsinki, P.O.B. 64, 00014 Helsinki, Finland; 2 Finnish Geospatial Research Institute, Geodeetinrinne 2, 02430 Masala, Finland; 3 Institute of Physics and Technology, Ural Federal University, Mira Str. 19, 620002 Ekaterinburg, Russia

**Keywords:** Engineering, Materials science, Physics

## Abstract

The primary goal of this study is to localize a defect (cavity) in a curved geometry. Curved topologies exhibit multiple resonances and the presence of hotspots for acoustic waves. Launching acoustic waves along a specific direction e.g. by means of an extended laser source reduces the complexity of the scattering problem. We performed experiments to demonstrate the use of a laser line source and verified the experimental results in FEM simulations. In both cases, we could locate and determine the size of a pit in a steel hemisphere which allowed us to visualize the defect on a 3D model of the sample. Such an approach could benefit patients by enabling contactless inspection of acetabular cups.

## Introduction

Acoustic wave generation and detection on curved geometries is challenging. Contacting techniques need to be adapted to the sample shape to effectively transmit and receive acoustic energy. Coupling sound to a sample may be hard e.g. when it is small or when sample access is restricted^1^. Furthermore, contacting transducers modify the boundary conditions of the sample under inspection and they are prone to oscillation^2^. As a result, both transmitted and received signals are distorted which make it hard to analyze the sample geometry. Practical difficulties arising from curvature include geometric focusing, and dispersion of acoustic waves. Acoustic waves generated on a spherical surface converge at the antipodal point. Measuring close to this focal point is desirable in order to achieve a high signal-to-noise ratio. The drawback in this approach is the presence of large amplitude gradients making the choice of exact measurement location critical to obtain repeatable results. Any recorded echo is masked by resonances since spherical structures are resonant geometries for acoustic waves^3^. Another unique feature of closed geometries is the presence of whispering gallery waves also known as circumferential waves. Circumferential waves, commonly used for pipe and cylinder inspection^4–8^, circumnavigate the sample multiple times and are characteristic of pipes and spheres^3, 9–12^. Compared to spheres, hemispheres add complexity to the interpretation of wave propagation since acoustic waves reflect off their equator. The aim of this study is to develop a method to visualize damage on a hemispherical shell.

Damage detection in shells is commonly done with Lamb waves. Their wave mode profile is defined with respect to the mid-plane between the two boundaries^13–19^. Any tapering^20^ or curvature of the plate^21–24^ modifies the symmetry plane of the particle displacement which alters the shape and cutoff frequencies of the acoustic waves. Such modified Lamb waves are sometimes called quasi Lamb waves^21^. Here we generate and detect Lamb waves by laser ultrasound in a contactless manner. Laser ultrasonic methods can efficiently generate and detect acoustic waves in a rapid manner with high bandwidth and spatial resolution^2^. Specific sample regions can be targeted by launching directed acoustic waves with extended laser sources^22, 25, 26^. This practice is commonplace for inspection of pipes^5, 8, 27, 28^ and Lamb waves are commonly used to characterize spherical shells^29^. Here we show an approach that benefits from a directed acoustic source to localize damage on a hemispherical shell.

Previously we showed that an extended line source combined with interferometric detection can locate holes as small as (∼0.7λ) on steel shells^18^. Here, we demonstrate how the acoustic field interacts with the cavity and we use this information to visualize the hole in a 3D model of the sample. This approach removes the need for complex scanning techniques^30^ and data interpretation based on mode conversion^31^. Such a simple approach could assist in the inspection of acetabular cups by characterizing their adhesion as well as the integrity of their attachment points^32–34^.

## Experiments

**Figure 1:**
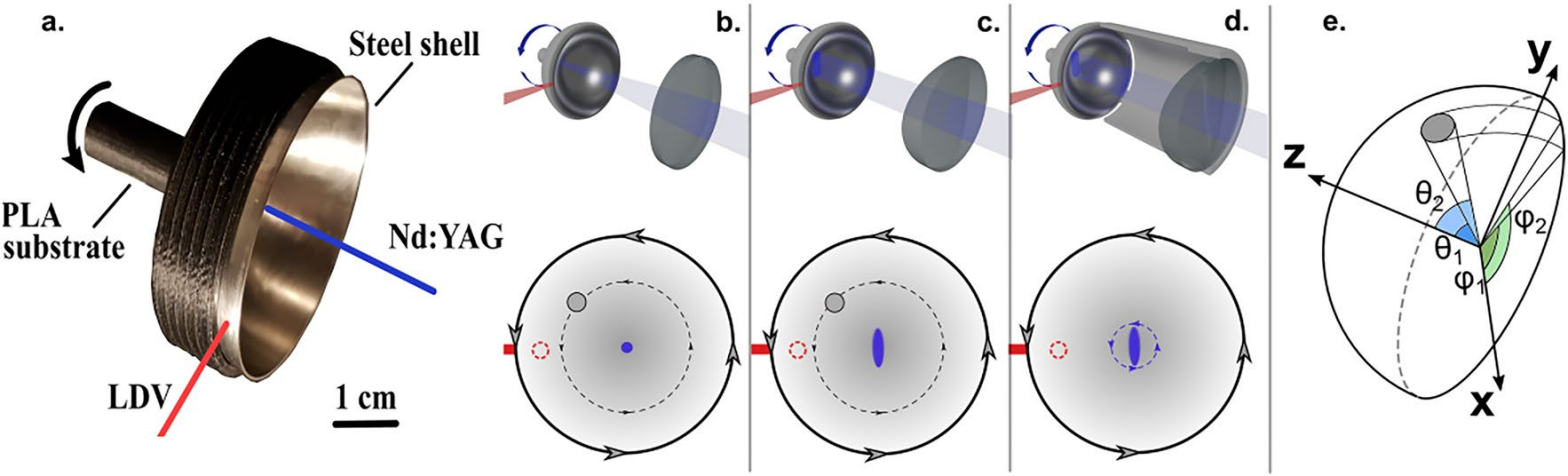
Experimental setup for laser ultrasound measurements. Nd:YAG laser (blue) generates guided waves on the sample whilst the laser Doppler vibrometer (LDV, red) detects them from the edge (**a**). The top view (bottom half) illustrates how the sample and the defect (grey circle) rotate in relation to the LDV (dashed red circle) and the Nd:YAG excitation laser (blue). A spherical (cylindrical) lens generates an acoustic point (line) source at the apex of the sample, (**b**–**d**). For localization (**e**), an effective approach is to use a stationary line source (**c**). We compare the acoustic directivity of the point and line sources on the hemispherical shell in the supplementary material of this study.

We developed the damage detection method by studying four stainless steel hemispherical shells (Ø = 50 mm and 0.6 mm thickness). These structures featured holes with diameters of 9.5 mm, 4 mm, and 2 mm at a polar angle θ = 45° whereas the last shell was pristine and served as a reference. We glued the samples to a 3D printed polylactic acid (PLA) holder with epoxy (Araldite® extra strong). The entire outer surface of the shell was attached to the holder except for a 3 mm wide brim (Fig. 1).

**Figure 2:**
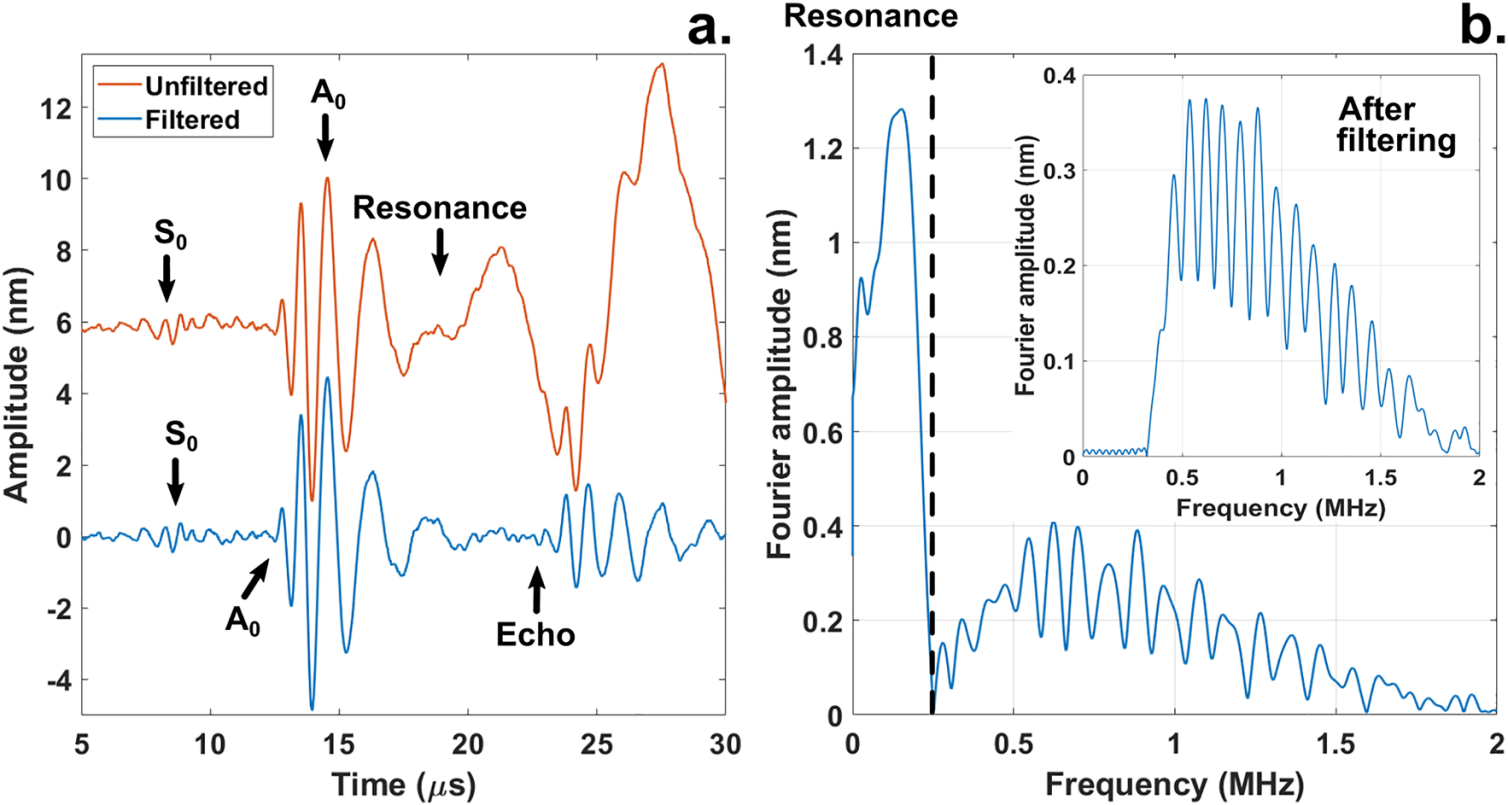
Filtering to remove standing waves in the structure. Signal shape produced by a point excitation on a damaged sample (**a**) and its frequency content (**b**). The raw signal (a, red, offset 6 nm for clarity) features a resonance that masks the echo. A high pass Fourier filter ranging from 0.1 to 0.5 MHz (**b**) removes the standing wave interference and reveals an echo (a, blue).

The hemispherical shell completed three rotations (200 points per revolution, 1.8° per step) under non-ablative pulsed illumination by a Q-switched Nd:YAG laser (CFR Big Sky Laser Series, 8 ns, 40 mJ per pulse, 3 Hz pulse repetition frequency, 12 pulses per step) whilst a Polytec OFV303 (OFV3001 controller) laser Doppler Vibrometer (2 MHz bandwidth in displacement mode) detected the acoustic waves at 0.8 mm distance from the edge of the sample. This approach generates acoustic maps by stacking signals of consecutive measurements next to each other. MATLAB [MathWorks® R2018b] generated the acoustic maps by averaging 12 signals at every point across the three rotations. To remove resonances which mask the acoustic waves, we filtered the data using a Butterworth high-pass filter (sampling frequency 62.47 MHz for experiments and 100 MHz for simulations, stop frequency = 0.1 MHz, pass frequency = 0.5 MHz). This approach reveals echoes carrying information about scatterers present in the structure (Fig. 2).

To localize damage on a 3D model of the hemisphere, we calculated the group velocity of the ${\mathrm{A}}_{0}$ mode. An algorithm extracted the time-of-arrival of the A_0_ mode from the data shown in Fig. 5b,e by finding the first peak of the guided wave front (Fig. 5c,f). For every azimuth we calculated the acoustic power and determined the -3 dB points (Fig. 5). These points delimit the boundaries of the pit of azimuthal coordinates φ_1_ and φ_2_ (Fig. 1e). We calculated the polar coordinates θ_1_ and θ_2_ from the propagation time of the echoes reflected from the defect at φ = 102.8° and φ = 295.2° (Fig. 5c,f). The calculation of the width and length of the defect when knowing the polar and azimuthal coordinates is straightforward and it is described in the calculations section of the supplementary material (Supplementary Fig. S2).

## Simulations

We recently did finite element method (FEM) simulations (COMSOL Multiphysics® v. 5.3a) of laser-generated ultrasound on a hemispherical shell^35^. We studied guided waves generated by a point source, their propagation on an intact sample, and their interaction with a circular defect. In the present study we expand our simulations to include the line excitation. Videos of the FEM simulations are provided in the supplementary material to this work.

The simulation models were built the same way as in^35^ for a 3D structure. COMSOL’s Heat Transfer Module was coupled to the Solid Mechanics Module through thermal stresses in the time domain to generate propagating elastic waves. Both the point and line excitations were modelled as Boundary Heat Sources with profiles corresponding to the ones used in the measurements. The boundary sources were defined as two perpendicular Gaussian profiles. The line source had FWHMs of 0.90 mm (line width) and 7.8 mm (line length), and the point source FWHM was 1 mm.

We determined the normal displacements at the surface of the sample at 1.85 mm distance from its edge and we assumed a point-like detection.

## Results

**Figure 3:**
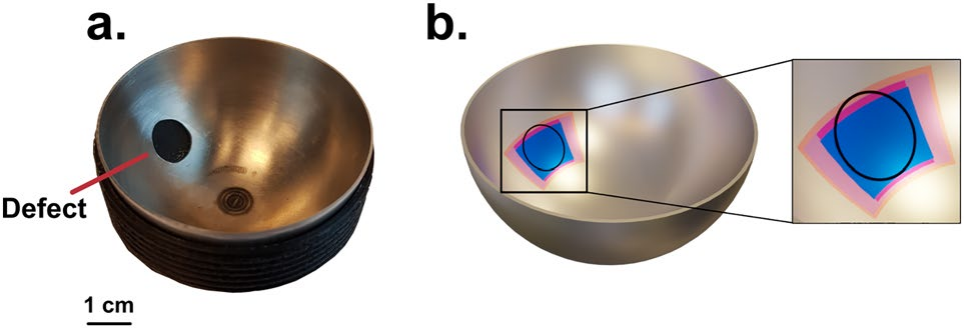
Comparison of a sample featuring a cavity (**a**) and the 3D model showing the localized defect (**b**). The blue and red regions indicate the size and location of a 9.5 mm defect predicted by the FEM simulations and the experimental results respectively. The translucent blue and red regions highlight the confidence limits of the defect size obtained from the simulations and the experiments respectively. The small mismatch in the polar direction might have been caused by a small offset produced when drilling the cavity.

**Figure 4:**
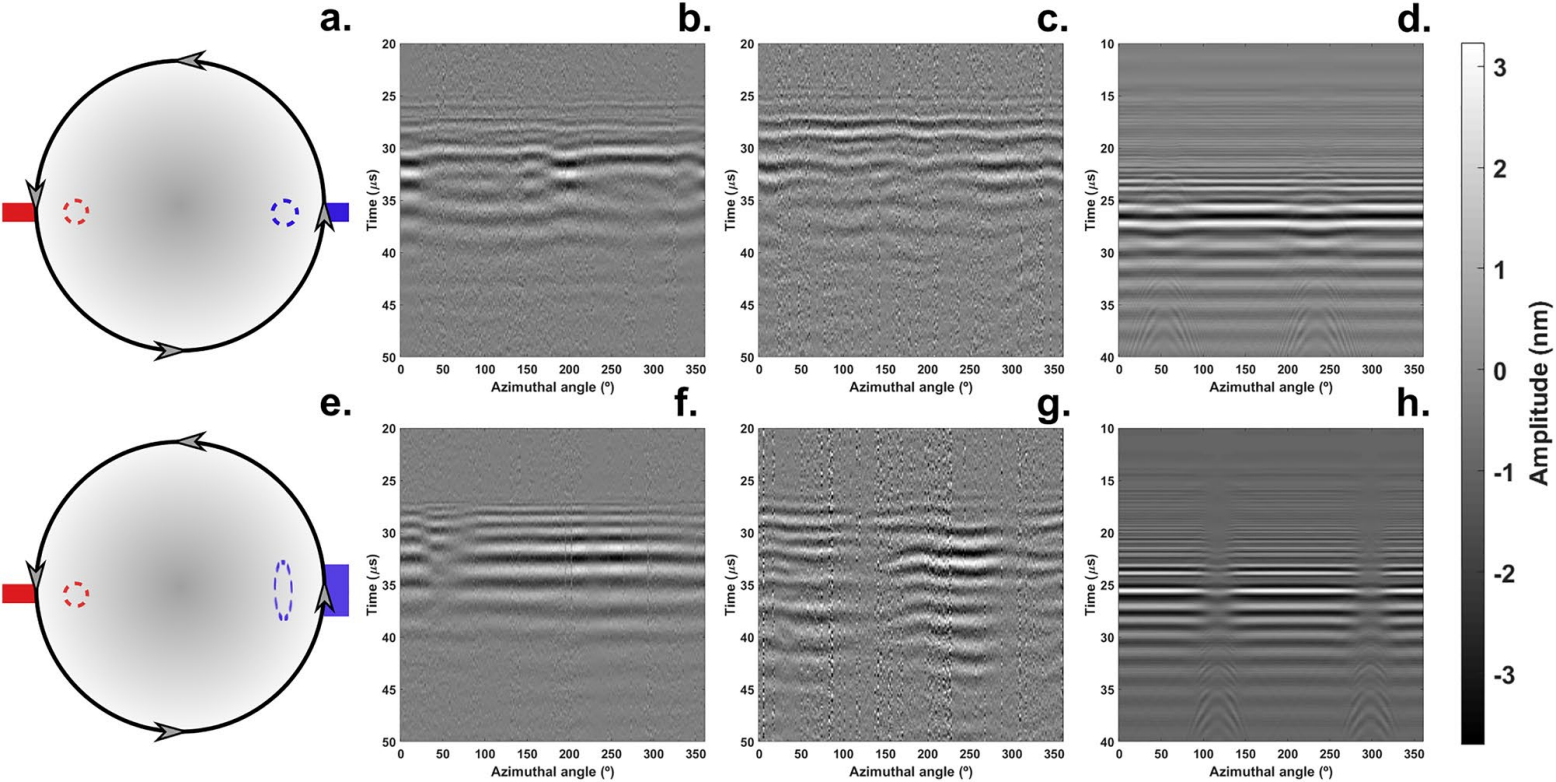
Detection of Lamb waves originated at the equator of the hemisphere. The Nd:YAG laser (dashed blue ellipse) generated acoustic waves from the edge of the hemisphere and the LDV aimed at the opposite end (dashed red circle) measured the vibrations (**a**,**e**). The acoustic maps consist of 200 signals stacked next to each other where the x-axis represents the azimuthal angle, the y-axis the propagation time of the acoustic waves and the color gradient the wave amplitude. Upper row: Lamb waves generated with a point laser source; Bottom row: Lamb waves generated with a laser line source. Intact sample (**b,f**); damaged sample with a 9.5 mm pit, experimental results (**c,g**) and FEM simulations (**d,h**). Data have been filtered to remove resonances. The laser line source produces a planar wave front whose shadowing (**g,h**) highlights the presence of a defect. In this configuration, there are insufficient data for calculating the polar angles since the echoes emanating from the defect are not visible in the experimental context (**g**).

**Figure 5:**
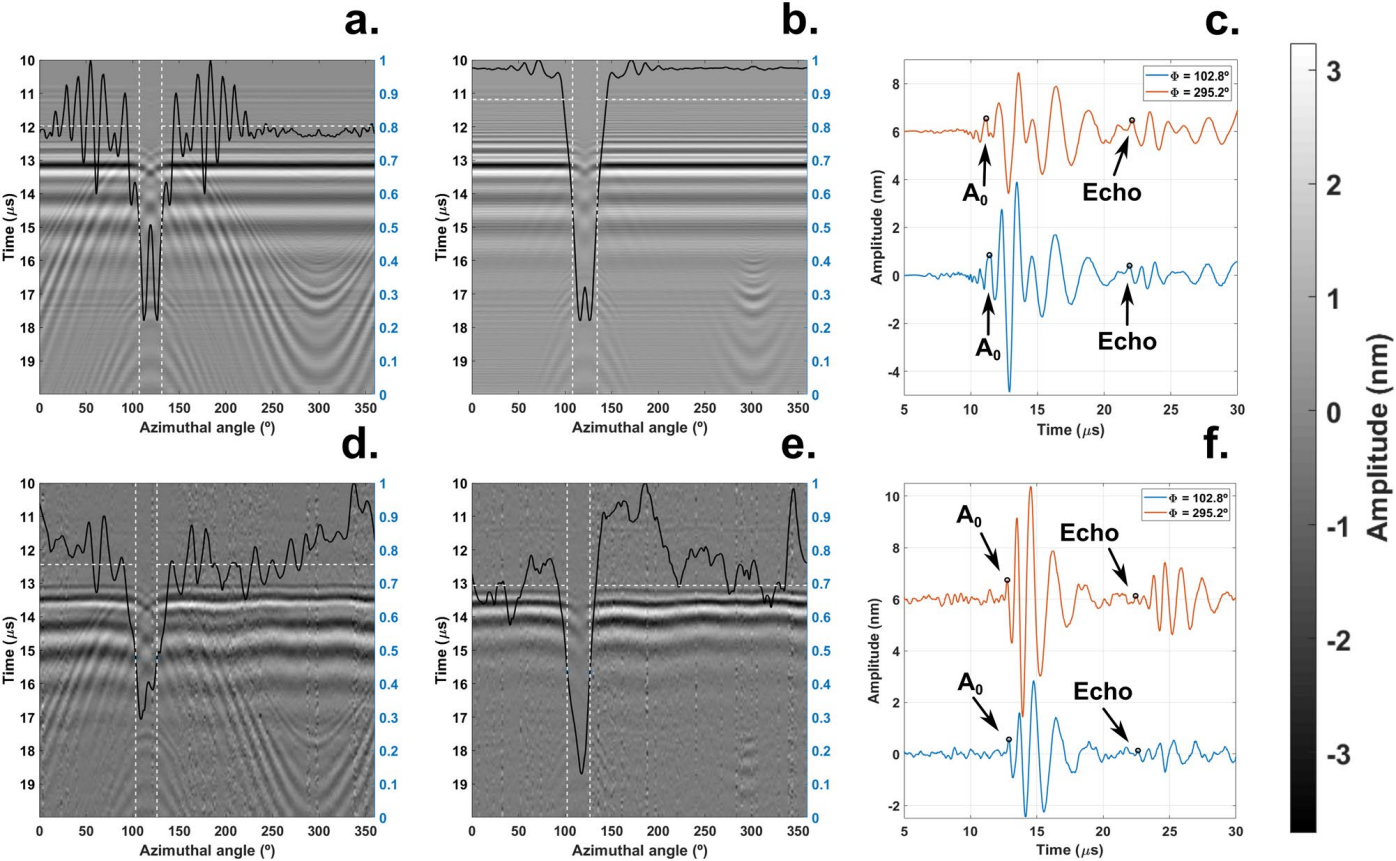
Detection of Lamb waves originated from the apex of the hemisphere. The acoustic maps (**a,b,d,e**) consist of 200 signals stacked next to each other where the x-axis represents the azimuthal angle, the y-axis the propagation time of the acoustic waves and the color gradient the wave amplitude. First row: Experimental results; second row: simulation results (a,d point source, b,e line source). c,f: echoes arising from a 9.5 mm defect on a laser line source scan at φ = 102.8° and its antipodal location φ = 295.2°. The black lines in (**b,e**) represent the acoustic power at different azimuths. The horizontal white lines represent its mean value across the undamaged region while the vertical lines are the −3 dB points. Data have been filtered to remove standing waves.

To localize the cavity on a 3D model of a hemisphere (Fig. 3), we first launched and detected the acoustic waves from two points on the equator of the sample (Fig. 4). We chose this configuration due to ease of access, however this approach did provide insufficient information for calculating the polar angles of the defect. Instead, we placed the detection point on the equator and the excitation location at the apex of the hemisphere. This approach yielded acoustic maps (Fig. 5) showing echoes radiating from the scatterer which allowed us to estimate the defect size and location (Table 1).

**Table 1:**
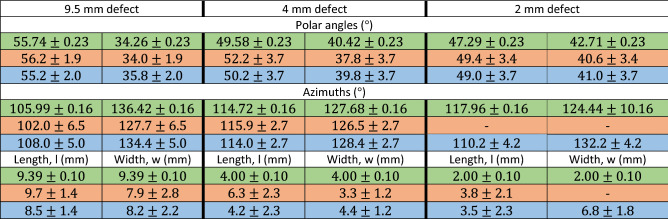
Defect location and size in the true location, simulated, and experimental cases for the 9.5 mm, 4 mm and 2 mm holes. From top to bottom, expected (green), experimental (orange), and simulation results (blue). Length and width are the dimensions of the sector containing the defect along both, the azimuthal and polar directions (Fig. 1e). The azimuthal coordinates as well as the width for the 2 mm defect in the experimental case are missing since there was no visible shadowing of the acoustic waveforms by the cavity (Supplementary Fig. S3). The magnitudes feature confidence limits of one standard deviation. The corresponding uncertainty calculations are presented in the supplementary material.

## Discussion and conclusions

The defect localization (Fig. 3) is made possible by the extended laser source. Such an acoustic source generates a planar wave package whose shadowing by a defect allows one to calculate the width of the region containing the scatterer. The width of the smallest detectable defect is determined by the size of the line source and the size of the scanning steps. In practice, this technique is applicable down to defect sizes equal to half the width of the acoustic source (3.9 mm). This has been verified both numerically and experimentally by studying defects of sizes down to 2 mm^18^ (Table 1, Supplementary Fig. S3). Each scanning step corresponded to an azimuthal rotation of 1.8° or 0.6 mm at the defect location. The calculation of the length of the defect can be frustrated by the long tails of the antisymmetric Lamb waves mode (Fig. 2). In our case, the tail from the direct wave would cover the echo radiating from the defect if the distance from the detection point to the scatterer would be smaller than 3 mm. Beyond these limitations another fundamental limiting factor is the wavelength of the interferometer. The OFV303 operated in displacement mode (2 MHz bandwidth) and considering the group velocity of the antisymmetric mode (Supplementary Eq. S2), the detectability limit is roughly 1.5 mm.

A challenge of using laser interferometry for damage localization is laser alignment. To achieve a high signal-to-noise ratio (SNR), the laser beam from the LDV must be perpendicular to the surface of the sample. The shortest optimum distance for the OFV303 is 232 mm. The leverage caused by this distance means that a 11.4’ tilt in the laser beam translates as a 1 mm distance on the surface of the sample. This is the reason why the distance from the LDV to the brim of the hemisphere is different for simulations and experiments. We performed multiple experiments to properly align the LDV and obtain a high SNR but decided not to repeat the simulations since they are computationally heavy taking about a month of computer time in total.

In this study, the radius of the hemisphere is much larger than the central acoustic wavelength λ ∼ 3 mm. Therefore, the impact of curvature on wave modes is negligible and we treat these vibrations as Lamb waves. The uniqueness of the problem arises when the excitation and the detection locations lie on the equator at diametrically opposite locations (Fig. 4). This region comprises a convergence point for acoustic waves, a point that features large gradients in the acoustic field amplitude (Supplementary Video 1). Consequentially, even small deviations in detection position make the experiment challenging to reproduce. Furthermore, our FEM simulations show that due to the reflections from the equator at these wavelengths, there is a region where the surface displacement is a node yielding low signal-to-noise ratio (Supplementary Fig. S2). We alleviated these issues by selecting the apex of the hemisphere as the source of acoustic waves while keeping the detection location on the brim of the hemisphere (Fig. 5).

Inspection of spheres and hemispheres suffer from interference caused by infinite paths of the same length connecting the poles on the shell surface. Path selection by directed acoustic fields minimizes the multipath interference. We further reduced this problem by moving the excitation location to the apex of the dome since, in the case of hemispheres, multipath interference only emerges when the detection and the excitation locations lie on the equator (Fig. 4).

As explained by Shui^3^, spheres are inherently resonant geometries for acoustic waves because of geometric focusing. When acoustic attenuation is low, acoustic waves converge at the antipodal point and circulate the surface of the hemisphere multiple times (Supplementary Video 2). Knowing the group velocity of the A0 mode, the resonance frequency is approximately ${f}_{res}=\frac{{c}_{A0}}{R}$ where ${c}_{{A}_{0}}$ and R are the group velocity of the A0 mode and R the radius of the hemisphere. In our case ${f}_{res}=120 \,\mathrm{kHz}$ which is close to the observed value ${f}_{res}=162\, \mathrm{kHz}.$ One could incorrectly interpret resonant modes as propagating waves leading to wrong conclusions. Filtering the data reveals an echo hiding under the resonance (Fig. 2). Knowing the time-of-flight of the echo and its angular width allowed us to estimate the size of the defect as well as its location (Table 1).

Despite the limitations of the introduced technique, the proposed approach extracts enough information to localize a defect in a 3D model of the sample (Fig. 3). Our method localized damage in curved geometries while minimizing interference caused by curvature. The contactless nature of the technique preserves the information of the propagating wave front since the sample boundaries are not loaded by contacting transducers^2^. Launching acoustic waves with an extended line source allowed us to localize a defect without the need of mapping the acoustic field across the surface of the sample^36^. The inspection of curved structures with buried defects could benefit from these advantages since it could be possible to image defects despite access being restricted^29, 32^.

## Supplementary Information

Supplementary Information 1.

Supplementary Video 1.

Supplementary Video 2.
