# Supplementary material for: Defect localization by an extended laser source on a hemisphere
Source: Sci Rep. 2021 Jul 26;11:15191. doi: 10.1038/s41598-021-94084-w (PMC8313693; doi:10.1038/s41598-021-94084-w)
Supplement: Supplementary file 1 — Supplementary Information 1. [file 41598_2021_94084_MOESM1_ESM.docx]

**Defect localization by an extended laser source on a hemisphere.**

Daniel Veira Canle^1,*^, Joni Mäkinen^1^, Richard Blomqvist^1^, Maria Gritsevich^1,2,3^, Ari Salmi^1^, Edward Hæggström^1^

^1^ Department of Physics, Division of Material Physics, Faculty of Science, P.O.B. 64, FIN-00014 University of Helsinki, Finland.

^2^ Finnish Geospatial Research Institute, Geodeetinrinne 2, 02430, Masala, Finland.

^3^ Institute of Physics and Technology, Ural Federal University, Mira str. 19, 620002 Ekaterinburg, Russia, PO Box. 56, Ekaterinburg, Russia.

*) Corresponding author:

Department of Physics

University of Helsinki

P.O.B. 64

00014 Helsinki

E-mail: [daniel.veiracanle@helsinki.fi](mailto:daniel.veiracanle@helsinki.fi)

Telephone: +358 4498 99903

Impact of probe beam location on signal-to-noise ratio.

To estimate the impact on the waveforms of a small distance offset of the LDV from the equator we performed the FEM simulations shown on Fig. S1. Here we studied the waveforms generated by a laser line source perpendicular to the detection spot. We moved the detection spot from the equator in steps of 0.25 mm from the edge along the polar direction.


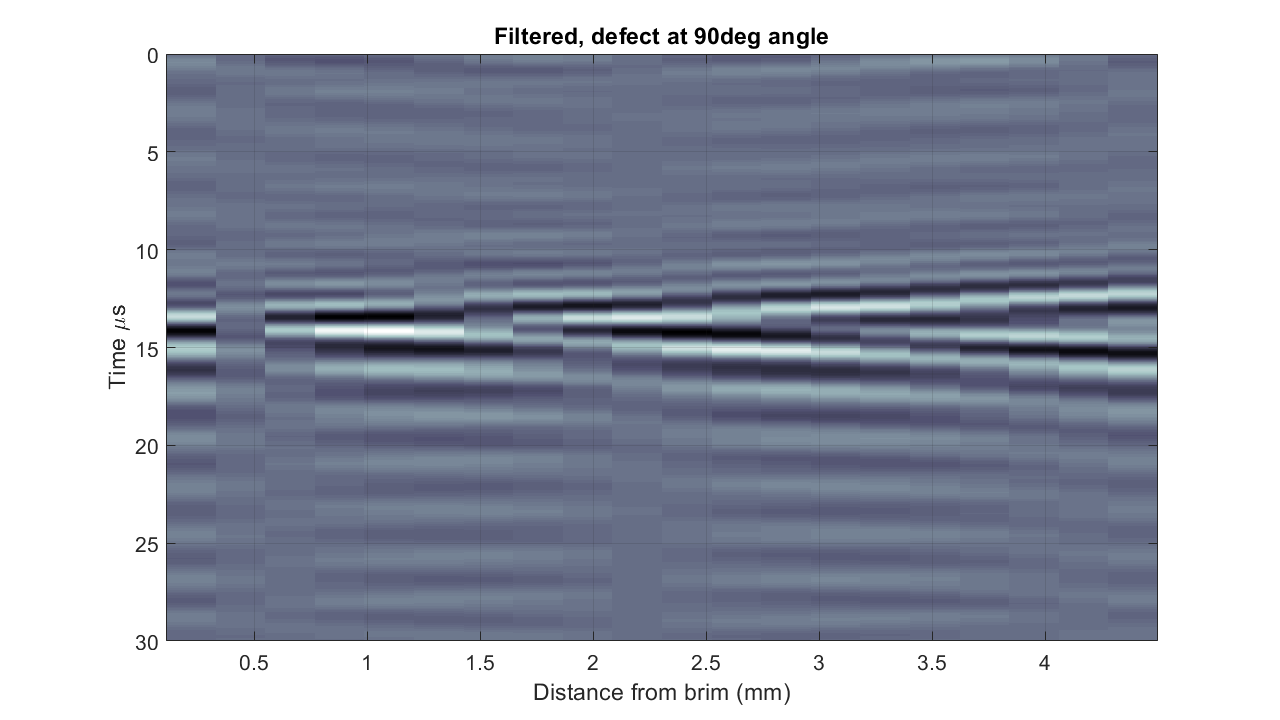

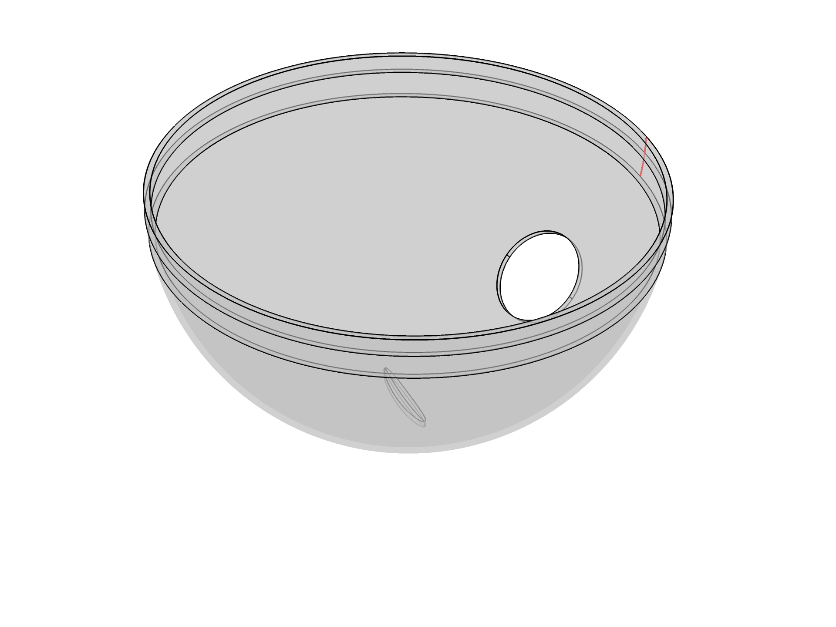


**Fig. S1. Impact of probe beam location on signal-to-noise ratio.**  Left: FEM simulation results show the change in amplitude as the LDV detection spot moves away from the equator of the hemisphere featuring a 9.5 mm defect. We hypothesize that at 0.5 mm there is a node caused by the superposition of multiple propagating waves reflected from the equator. Consequently, the signal-to-noise ratio is low in this region. Right: Representation of the 3D model used for FEM simulations (left). The laser line excitation is visible at the apex of the hemisphere and the LDV scanning path is highlighted in red.

Estimation of defect size and location

To reconstruct the acoustic field scattered by a cavity on a 3D model of the hemisphere, we calculated the group velocity of the $A_{0}$ mode (Eq. 1). An algorithm extracted the time-of-arrival of the A_0_ mode from data by finding the first peak of the guided wave front (Fig. 5C, 5F).

$$v_{A_{0}}=\frac{d}{t}=\frac{1}{t}\left( \frac{\pi}{2}R-0.85 mm \right)=\left( 3056\pm123 \right) ms^{-1} (1)$$

Here, *d* is the arc length between the excitation and the detection point, *t* is the time-of-flight of the $A_{0}$ mode, and *R* is the radius of the hemisphere.

Making a hole in a steel hemisphere results in a region with equal length *l* and width *w* according to (Eq. 2):

$$w=l=\alpha R=R\mathrm{atan} \left( \frac{d}{2R} \right) \alpha=\mathrm{atan} \left( \frac{d}{2R} \right) (2)$$

where *d* is the hole diameter and α the angle describing the boundaries of the defect. We used this information to calculate the expected location of the defect boundaries. Since we used a jig to create the hole, we know that the center of the damaged region is at a polar angle $\theta$ = 45º. We positioned the setup so that the flaw was centered at an azimuth $\phi=121^{\circ}$. Therefore, the expected coordinates are $\theta_{1,2}=45^{\circ}\pm\frac{\alpha}{2} \phi_{1,2}=121\pm\frac{\alpha}{2}$.

To determine the azimuth at which the damage is located we calculated the power across the wave front (Eq. 3).

$$P \alpha\frac{\int_{t_{1}}^{t_{2}} {A\left( t \right)}^{2}dt}{\int_{t_{1}}^{t_{2}} dt} \mathrm{where} t_{1}=10 \mu s \mathrm{and} t_{2}=20 \mu s (3)$$

We hypothesize that the acoustic power *P* is proportional to the amplitude squared *A^2^* of the propagating guided wave. These waves interact with defects by scattering and the detected acoustic power is reduced. In the cases dealt with in this study, the ka number (where k is the wave number and a the radius of the particle) ranges from 2 to 10. Since the size of the defect is much larger than the wavelength, the scatterer is non-selective. The regions where the power drops by -3 dB determine the azimuthal coordinates of the defect $\phi_{1}$, $\phi_{2}$. These angles yield the width *w* of the region containing the defect (Eq.4).

$$w=r \left( \phi_{2}-\phi_{1} \right)=R\sin\left( \frac{\pi}{4} \right)\left( \phi_{2}-\phi_{1} \right) (4)$$

Here r is the radial distance in the spherical reference system $r=R\sin\left( ϴ \right)=R\sin\left( \frac{\pi}{4} \right)$ and it is defined at the defect location.


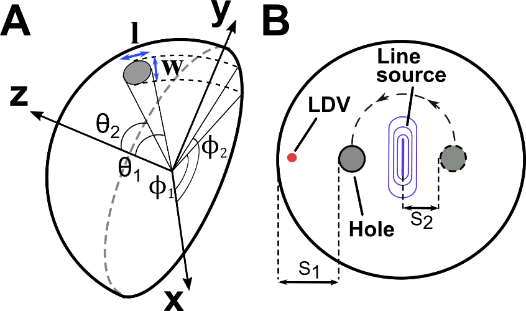


**Fig. S2. Coordinate system and polar angle calculation.** A) The sector delimiting the defect (gray circle) is defined by the azimuths $\phi_{1}, \phi_{2}$ and the polar angles $\theta_{1}$, $\theta_{2}$. It features a width w and length l, and we do not consider the sample thickness.

The difference between the time-of-flight of the ballistic wave and the echo yields the distances $s_{1}$ and $s_{2}$. Knowing these values, the defect length *l* is (Eq.5):

$$s_{1,2}=\frac{\Delta t_{1,2}}{2}v_{A_{0}} \theta_{1}=\frac{1}{R}s_{1} \theta_{2}=\frac{\pi}{2}- \frac{1}{R}s_{2} (5)$$

$$l=\frac{\pi}{2}R-s_{1}-s_{2}$$

Here $\theta_{1}$ and $\theta_{2}$ are the polar angles depicted in Fig. S2, $v_{A_{0}}$is the $A_{0}$ mode’s group velocity and *R* is the outer radius of the hemisphere. $\Delta t_{1}$and $\Delta t_{2}$ are the time differences between the $A_{0}$ mode arrival and the time-of-flight of the corresponding echoes (Fig. S3).


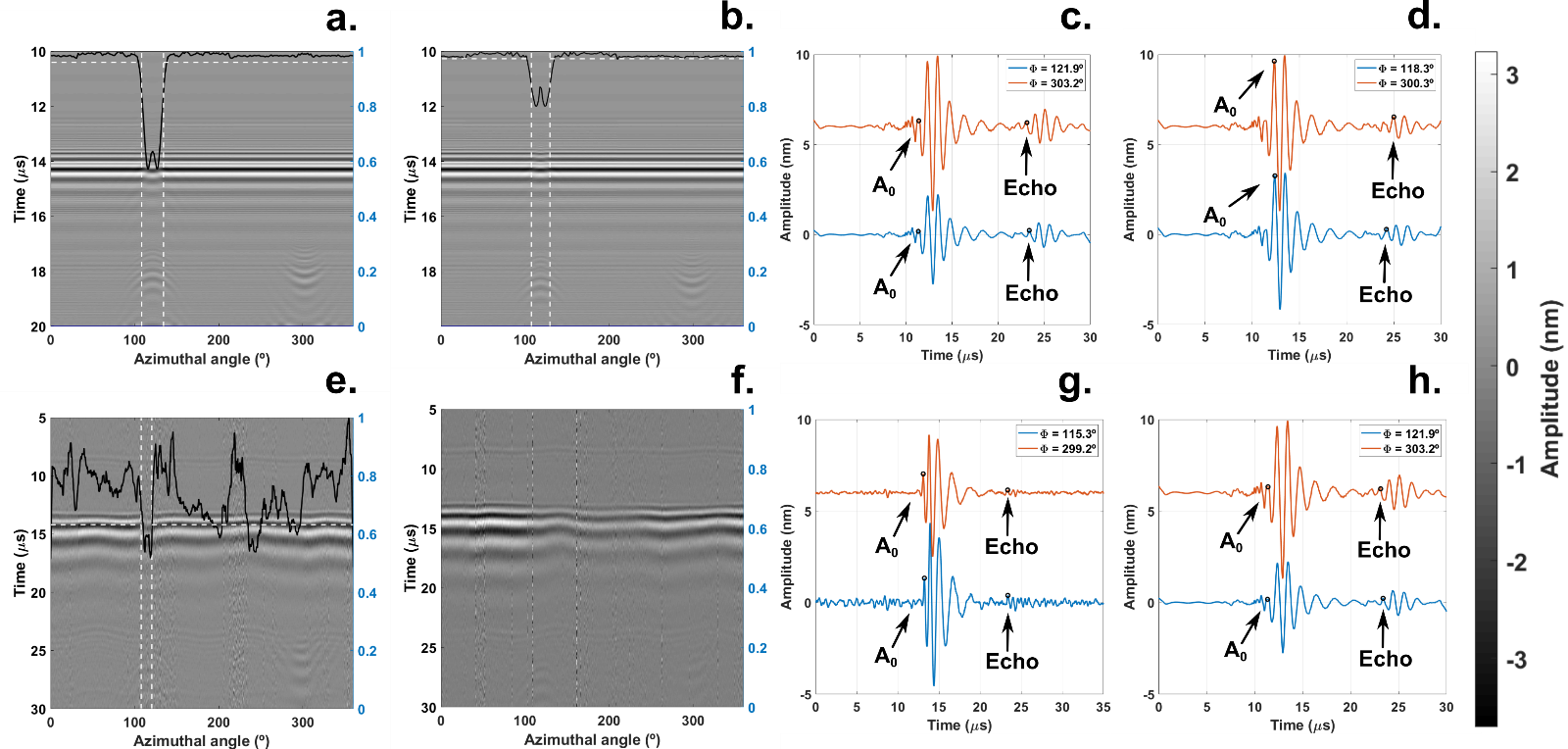


**Fig. S3. Detection of Lamb waves originated from the apex of the hemisphere with a laser line excitation.** The acoustic maps (a,b,e,f) consist of 200 signals stacked next to each other where the x-axis represents the azimuthal angle, the y-axis the propagation time of the acoustic waves and the color gradient the wave amplitude. First row: experimental results; second row: simulation results (first column: 4-mm defect; second column: 2-mm defect). The black lines in a, b and e represent the acoustic power at different azimuths. The horizontal white lines represent its mean value across the undamaged region while the vertical lines are the -3 dB points. The shadowing of the acoustic wave front is not visible in the experimental study of the 2-mm defect (f). To calculate the polar angles as well as the length of the sector containing the defect, we studied the waveforms shown in the third and fourth columns. These signals feature the ballistic A_0_ mode as well as the echo radiating from the 4-mm (c,g) and 2-mm (d,h) defect respectively.

Directivity study

We define directivity as the polar map of the acoustic power calculated from Eq.3. Experimentally, we compared the directivity of the acoustic field generated by a point and a line source on the surface of the intact hemisphere (Fig S4). Figure 1 shows the experimental setup. To determine the directivity produced by a point source, we used a spherical lens to focus the Nd:YAG laser into a spot (Ø=1 mm) at the apex of the shell (Fig.1B). The equivalent case for the line excitation required a cylindrical lens rotating with the sample. We inserted a cylindrical lens into a holder screwed into the sample which allowed focusing the excitation laser into a line (7.8 mm long, 0.8 mm wide) at the apex (Fig. 1D). The screw-on adaptor featured windows allowing the vibrometer to detect acoustic waves at the brim of the hemisphere.

In the FEM realm to simulate a scan with a fixed line excitation only one simulation is needed. The acoustic energy directivity study requires a rotating line source. Due to the spherical symmetry of the problem we simulated a 90° rotation of the line excitation instead of a 360º rotation. To achieve this, two points located along the line at 180º from each other detected the wave amplitude normal to the sample surface. Mirroring the scan results yielded a 360º map of the propagating waves in the hemispherical structure (Fig S4).


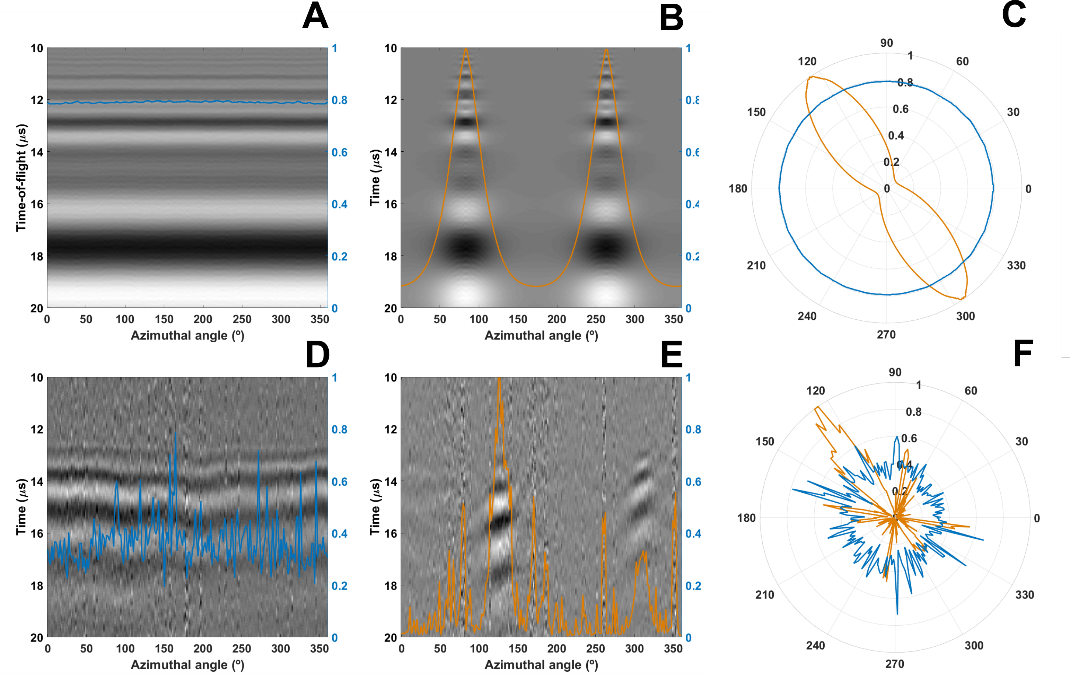


**Fig. S4. Comparison of the acoustic field created by a point and a line source on the surface of an intact hemispherical shell as depicted in Fig.1b&d.** First row: simulation results; second row: experimental results; first column: point source; second column: line source; third column: directivity maps. The orange and blue lines correspond to a point and line excitation respectively and have been calculated according to Eq.3. These values have been normalized to the maximum of their respective line source excitation.

Uncertainty calculation and analysis

With a digital caliper we measured the sample’s outer diameter, thickness, and distance from the LDV detection point to the edge. We assumed that these magnitudes contribute with a similar magnitude of uncertainty since they were measured with the same instrument featuring a resolution $S\left( d \right)=0.05 mm$. In the following analysis, we assumed that the magnitudes are uncorrelated since they are independent entities.

The uncertainty of the fastest $A_{0}$ group velocity (Eq. 6) is

$$S\left( v_{A_{0}} \right)=\frac{1}{t}\sqrt{S^{2}\left( d \right)+v^{2}S^{2}\left( t \right)} (A1)$$

Here $S^{2}(d)$ and $S^{2}(t)$ are variance in path and time-of-flight, respectively. Since *t* is the time-of-flight average of the $A_{0}$ mode:

$$S\left( t \right)= \sqrt{S^{2}\left( t_{\exp} \right)+S^{2}(t_{\mathrm{av}})} (A2)$$

$S(t_{\exp})$ is the time difference between the second and first peaks of the $A_{0}$ wave front. $S(t_{\mathrm{av}})$ is the standard deviation of the mean. This is the result of averaging the time-of-flight of the first peak of the $A_{0}$ mode.

We estimated the uncertainty in the polar angle calculation as:

$$S\left( ϴ \right)=\frac{1}{D}\sqrt{v_{A_{0}}^{2}S^{2}\left( \Delta t \right)+ \Delta t^{2}S^{2}\left( v_{A_{0}} \right)+ ϴ^{2}S^{2}(D)} (A3)$$

Here $S(ϴ)$ is the standard deviation of the polar angle, $S^{2}(\Delta t)$ is the variance of the time difference between the time-of-flight of the echo radiating from the defect and the ballistic wave. $S^{2}(D)$ is the variance of the sample diameter *D* being $S\left( D \right)=S\left( d \right)=S\left( R \right)=0.05 mm$.

The uncertainty in defect length is:

$S\left( l \right)=\sqrt{S^{2}\left( s_{T} \right)+S^{2}\left( s_{1} \right)+S^{2}\left( s_{2} \right)}=\sqrt{S^{2}\left( s_{T} \right)+2S^{2}\left( s \right)}$ $(A4)$

The uncertainty in the azimuth arises from the acoustic power calculation. Here we estimated $S(\phi)$ as the angle difference between the average power level and the -3 dB point (Fig 6B, 6E).

From Eq. 3 using propagation of uncertainties yields:

$$S\left( w \right)= \sqrt{\left( \Delta\phi\right)^{2}S^{2}\left( R \right)+{R^{2}S}^{2}\left( \Delta\phi\right)}\sin\left( \frac{\pi}{4} \right) (A5)$$
